# Supplementary material for: Identification and functional analysis of non-coding regulatory small RNA FenSr3 in Bacillus amyloliquefaciens LPB-18
Source: PeerJ. 2023 May 15;11:e15236. doi: 10.7717/peerj.15236 (PMC10194069; doi:10.7717/peerj.15236)
Supplement: Supplemental Information 4 [file peerj-11-15236-s004.zip › KO/CK-vs-T1_map/map05217.html]

KEGG PATHWAY: Basal cell carcinoma - Reference pathway


|  |  |
| --- | --- |
| **Basal cell carcinoma - Reference pathway** |  |

[
Pathway menu
| Organism menu
| Pathway entry
| Show description
| User data mapping
]

Cancer of the skin is the most common cancer in Caucasians and basal cell carcinomas (BCC) account for 90% of all skin cancers. The vast majority of BCC cases are sporadic, though there is a rare familial syndrome basal cell nevus syndrome (BCNS, or Gorlin syndrome) that predisposes to development of BCC. In addition, there is strong epidemiological and genetic evidence that demonstrates UV exposure as a risk factor of prime importance. The development of basal cell carcinoma is associated with constitutive activation of sonic hedgehog signaling. The mutations in SMOH, PTCH1, and SHH in BCCs result in continuous activation of target genes. At a cellular level, sonic hedgehog signaling promotes cell proliferation. Mutations in TP53 are also found with high frequency (>50%) in sporadic BCC.

|  |  |  |
| --- | --- | --- |
| Reference pathway | 184% 150% 122% 100% 82% 67% 55% | 图片下载 |
